# Interfacial compatibility critically controls Ru/TiO_2_ metal-support interaction modes in CO_2_ hydrogenation

**DOI:** 10.1038/s41467-021-27910-4

**Published:** 2022-01-17

**Authors:** Jun Zhou, Zhe Gao, Guolei Xiang, Tianyu Zhai, Zikai Liu, Weixin Zhao, Xin Liang, Leyu Wang

**Affiliations:** 1 State Key Laboratory of Chemical Resource Engineering, Beijing University of Chemical Technology, Beijing, 100029 China; 2 State Key Laboratory of Coal Conversion, Institute of Coal Chemistry, Chinese Academy of Sciences, Taoyuan South Road 27, Taiyuan, 030001 China

**Keywords:** Heterogeneous catalysis, Catalytic mechanisms, Structural properties

## Abstract

Supports can widely affect or even dominate the catalytic activity, selectivity, and stability of metal nanoparticles through various metal-support interactions (MSIs). However, underlying principles have not been fully understood yet, because MSIs are influenced by the composition, size, and facet of both metals and supports. Using Ru/TiO_2_ supported on rutile and anatase as model catalysts, we demonstrate that metal-support interfacial compatibility can critically control MSI modes and catalytic performances in CO_2_ hydrogenation. Annealing Ru/rutile-TiO_2_ in air can enhance CO_2_ conversion to methane resulting from enhanced interfacial coupling driven by matched lattices of RuO_x_ with rutile-TiO_2_; annealing Ru/anatase-TiO_2_ in air decreases CO_2_ conversion and converts the product into CO owing to strong metal-support interaction (SMSI). Although rutile and anatase share the same chemical composition, we show that interfacial compatibility can basically modify metal-support coupling strength, catalyst morphology, surface atomic configuration, MSI mode, and catalytic performances of Ru/TiO_2_ in heterogeneous catalysis.

## Abstract (web summary)

Supports can largely affect the catalytic performance of metal nanoparticles, but the underlying principles are not yet fully understood. Here the authors demonstrate that metal-support interfacial compatibility of Ru/TiO_2_ can critically control the metal-support interaction modes and the catalytic performances in CO_2_ hydrogenation.

## Introduction

Supported metal nanoparticles (NPs) dominate practical catalysts in producing bulk and fine chemicals, reducing environmental emissions, energy conversions, etc.^1–3^. Their catalytic performances (activity, selectivity, and stability) not only depend on the composition, size, shape, and ligation state of metal NPs, but are also highly affected or even dominated by supports^4–11^. Metal-support interaction (MSI) has therefore become a central topic in heterogeneous catalysis^1,3,12,13^. Typical MSI modes include strong metal-support interaction (SMSI), interfacial charge transfer, interfacial perimeter, spillover, etc.^1^. In particular, SMSI phenomena, featuring in encapsulating metallic NPs (such as Pt, Au, Pd, Rh, Ru, and Ni) by reducible oxide supports like TiO_2_, can widely modify catalytic activity and selectivity of hydrogenation reactions^9,14–20^. Although various MSI forms have been reported, the underlying tuning principles still remain elusive, because all structural factors can affect MSIs, such as the composition, size, and shape of metals, the composition, phase, facet, and size of supports, as well as adsorbates and reaction atmospheres^1,12,21–25^. Moreover, their simultaneous interactions extremely complicate MSI phenomena and challenge the study on the mechanisms. Exploring the principles dominating support effects and MSIs is therefore crucial for rational design, optimization, and understanding of heterogeneous catalysis.

Among all structural factors, metal-support interface should play the primary role, because all MSI modes occur based on the direct contacts of catalysts with supports^26,27^. For solid–solid contacts, coupling strength is the most fundamental parameter determining the property and stability of their interfaces, which is thermodynamically described with adhesion energy (Φ_adh_)^28,29^. The relative value of Φ_adh_ to bulk cohesion energy (Φ_coh_) basically determines interfacial contact angles and thermal stability of supported particles^30^. In catalysis science, adhesion energy widely controls the morphologies and sintering rates of supported-metal NPs^31,32^. Many post-treatment methods such as thermal annealing and reduction–oxidation cycles can modify interfacial adhesion and catalytic performances^1,9,16,22,33,34^. Furthermore, at the atomic scale, interfacial coupling occurs through forming chemical bonds, thus, interfacial bonding strength basically determines metal-support adhesions^35^. For example, Campbell et al. theoretically studied the trends in the adhesion energies of metal NPs on various oxide surfaces, and found that higher metal oxophilicity and more active surface-oxygen atoms could lead to stronger metal-support adhesions^29^. Senftle et al. further revealed that interfacial binding strengths between single-metal atoms and oxide supports depended on the oxophilicity of supported metals and reducibility of oxide supports^36^. Despite these understandings on the surface stability of metal NPs on oxides, the structure–function relationships on how interfacial structure features modify MSI modes and catalytic performances are still not fully revealed yet^9,33,34,37^.

At the atomic scale, strong catalyst-support contacts result from interfacial bonds. The strength parameters, macroscale adhesion energy, and microscale bonding energy can be correlated following:

1 $${{\Phi}}_{{{{{\rm{adh}}}}}}=k{E}_{{{{{\rm{IB}}}}}}{N}_{s}$$

where *E*_IB_ and *N*_*s*_ denote the average energy and surface density of interfacial bonds, and *k* is a coefficient. Thus, Φ_adh_ can be enhanced by increasing *E*_IB_ or *N*_*s*_. Both *E*_IB_ and *N*_*s*_ further depend on the atomic configurations of contacting surfaces, because the positions of interfacial atoms intrinsically affect the length, angle, and density of interfacial bonds. Therefore, interfacial configurations of catalysts and supports are intrinsic structural factors controlling catalyst states and MSI effects. The matching degree of interfacial configurations is also referred to as interfacial compatibility, which measures the strength of interfacial bonding and adhesion^27,30,35^. Catalysts weakly wet supports at misfit interfaces with low interfacial compatibilities, which leads to ready phase separations at the interfaces and catalyst sintering. While high interfacial compatibility can increase both the strength and density of interfacial bonds^13,35^. The highest interfacial compatibility exists in epitaxial interfaces between lattice-matched materials. In general, lattice misfit less than 5% can form epitaxial overlayers with zero contact angles. This principle has widely guided the growth of semiconductor and oxide heterogeneous structures with minimized interfacial defects^38^. However, the mechanism of how metal-support contacts and their interfacial compatibilities affect MSI modes and catalytic performances has been rarely revealed yet.

**Fig. 1:**
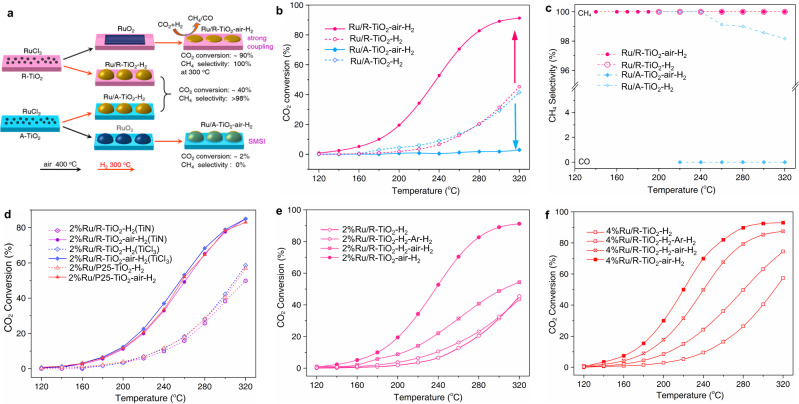
Opposite catalytic performances of Ru/TiO_2_ supported on rutile (R–TiO_2_) and anatase (A–TiO_2_) for CO_2_ hydrogenation. **a** Summary scheme of varied activity and selectivity by direct H_2_ reduction and after annealing in air. Ru/TiO_2_–H_2_ refers to directly reduced catalysts by H_2_, while Ru/TiO_2_–air–H_2_ refers to catalysts by annealing in air at 400 °C and further reduction by H_2_. **b** Temperature-dependent CO_2_ conversions. **c** Temperature-dependent CH_4_ selectivity. **d** Temperature-dependent CO_2_ conversions of 2% Ru/R–TiO_2_ catalysts on other R–TiO_2_ supports that were prepared from TiN and TiCl_3_, and P25 TiO_2_. Temperature-dependent CO_2_ conversions by annealing (**e**) 2% Ru/R–TiO_2_–H_2_ and (**f**) 4% Ru/R–TiO_2_–H_2_ catalysts in Ar and air. Ru/R–TiO_2_–H_2_–air–H_2_ means the catalyst was first reduced by H_2_ at 300 °C, then annealed in air at 400 °C, and reduced with H_2_ at 300 °C again.

Using Ru/TiO_2_ as a model catalyst, here we demonstrate the critical role of interfacial compatibility in controlling MSI modes, surface atomic states, and catalytic activity and selectivity in CO_2_ hydrogenation reaction. Rutile (R–TiO_2_) and anatase (A–TiO_2_) are used as supports to vary interfacial structures. RuO_2_ shares the same lattice structure with R–TiO_2_, which can lead to a high interfacial compatibility to form epitaxial overlayers^39^. The interfacial RuO_x_ species can act as anchoring layers to strengthen interfacial bonding between Ru and R–TiO_2_. For CO_2_ hydrogenation reaction, we find that both catalytic activity and selectivity can be oppositely modified by annealing Ru/TiO_2_ catalysts in air. On R–TiO_2_, Ru shows enhanced activity and dominant selectivity of CH_4_; on A–TiO_2_, Ru shows decreased activity and dominant selectivity of CO (Fig. 1a). Such opposite catalytic performances clearly indicate that interfacial compatibility can vary MSI modes—Ru/A–TiO_2_ shows normal SMSI effect, while Ru/R–TiO_2_ displays strong interfacial coupling.

## Results

### Support effects on activity and selectivity of Ru/TiO_2_ for CO_2_ hydrogenation

All Ru/TiO_2_ catalysts were prepared following the same protocol to minimize the interference from synthetic conditions. To stabilize the surfaces and prevent further sintering during catalyst preparations and reactions, the supports were first annealed in air at 500 °C for 10 h (Supplementary Fig. 1). The annealed supports and RuCl_3_ were uniformly mixed in water, rapidly frozen with liquid nitrogen, and dried in a freezing drier (Supplementary Fig. 2). The fully mixed RuCl_3_–TiO_2_ precursors were reduced by H_2_ at 300 °C either directly (Ru/TiO_2_–H_2_) or after pre-annealing in air at 400 °C (Ru/TiO_2_–air–H_2_). CO_2_ hydrogenation was conducted at normal pressure with gas hourly space velocity (GHSVs) of 12,000 mL·g^−1^·h^−1^, in which the reaction gas composed of 60 vol% H_2_/15 vol% CO_2_/25 vol% Ar. At normal pressure, the products of CO_2_ hydrogenation are CH_4_ and CO. Therefore, CO_2_ conversion denotes catalytic activity, and the ratio of n(CH_4_)/(n(CO) + n(CH_4_)) denotes selectivity, while the varied activity and selectivity further reflect different support effects and MSI modes^5,12,40–43^.

Annealing RuCl_3_–TiO_2_ precursors in air can effectively modify catalytic performances of Ru/TiO_2_ catalysts (Fig. 1a, b). Figure 1b presents temperature-dependent CO_2_ conversions by 2% Ru/TiO_2_. Ru/R–TiO_2_–H_2_ and Ru/A–TiO_2_–H_2_ show similar CO_2_ conversions between 120 °C and 320 °C, suggesting that R–TiO_2_ and A–TiO_2_ apply similar support effects on directly reduced Ru/TiO_2_ catalysts. However, the conversions dramatically differentiate by pre-annealing RuCl_3_–TiO_2_ precursors in air at 400 °C. Ru/R–TiO_2_–air–H_2_ displays an enhanced catalytic performance, with CO_2_ conversion at 300 °C increasing from 31.4% to 89.2%; Ru/A–TiO_2_–air–H_2_ shows a highly decreased activity, with CO_2_ conversion at 300 °C reducing from 29.4% to 1.7%. At each reaction temperature, Ru/R–TiO_2_–air–H_2_ shows the highest CO_2_ conversions among four catalysts. The results indicate that R–TiO_2_ and A–TiO_2_ apply opposite support effects on the activity of Ru NPs.

In addition to activity, annealing RuCl_3_–TiO_2_ precursors in air can also dramatically modify the catalytic selectivity of Ru on R–TiO_2_ and A–TiO_2_ (Fig. 1c). CH_4_ dominates both the products of Ru/R–TiO_2_–H_2_ and Ru/A–TiO_2_–H_2_ between 200 °C and 320 °C (>98%). However, the product on Ru/A–TiO_2_–air–H_2_ converts into 100% CO between 220 °C and 320 °C, while on Ru/R–TiO_2_–air–H_2_ is still 100% CH_4_ between 140 °C and 320 °C. The different products show another effect of supports on the catalytic performances of Ru NPs^18,41^.

The enhancement effect of air-annealing on the activity of Ru/R–TiO_2_ is a general trend. We verified the phenomena by varying the loading amounts of Ru, rutile supports, and post-processing procedures. The activities of 1% Ru/R–TiO_2_ and 4% Ru/R–TiO_2_ can also be enhanced by pre-annealing in air, showing the same trend with 2%-Ru/R–TiO_2_ (Supplementary Fig. 3). Moreover, the activities of 2% Ru/R–TiO_2_ supported on other R–TiO_2_ materials prepared using TiCl_3_ and TiN (Fig. 1d) as precursors, and P25 TiO_2_, a commercial TiO_2_ product composed of 4/5 anatase and 1/5 rutile, can also be effectively enhanced by pre-annealing in air. It is noted that CO_2_ conversions by Ru/R–TiO_2_–H_2_ and Ru/R–TiO_2_–air–H_2_ are almost the same on these three supports, confirming the stable reproducibility of this enhancement effect.

We further annealed 2% Ru/R–TiO_2_–H_2_ (Figs. 1e) and 4% Ru/R–TiO_2_–H_2_ (Fig. 1f) catalysts at 400 °C in 25-sccm air or Ar flows, and then reduced with H_2_ at 300 °C. CO_2_ conversion by the annealed catalyst in Ar (2% Ru/R–TiO_2_–H_2_–Ar–H_2_) is similar to that of 2% Ru/R–TiO_2_–H_2_. In contrast, CO_2_ conversions by the annealed catalyst in air (2% Ru/R–TiO_2_–H_2_–air–H_2_) notably increase between 160 °C and 320 °C. In particular, CO_2_ conversion increases from 1.9% to 8.8% at 200 °C, and 31.5% to 48.7% at 300 °C. This promotion effect can also be supported by the reduced apparent activation energies (*E*_*a*_, Supplementary Fig. 4). *E*_*a*_ of 2% Ru/R–TiO_2_–H_2_ is 66.5 kJ·mol^−1^, while the values are 52.3 kJ·mol^−1^ and 40.4 kJ·mol^−1^ for 2% Ru/R–TiO_2_–H_2_–Ar–H_2_ and 2% Ru/R–TiO_2_–H_2_–air–H_2_, respectively^5^. The enhancement effect on 4% Ru/R–TiO_2_–H_2_ is more apparent than 2% Ru/R–TiO_2_ between 140 °C and 320 °C after annealing in Ar and air. This is because Ru nanoparticles on 4% Ru/R–TiO_2_–H_2_ are larger, and annealing can more effectively increase their contacts with R–TiO_2_ supports.

### Geometric states of Ru NPs on TiO_2_ supports

**Fig. 2:**
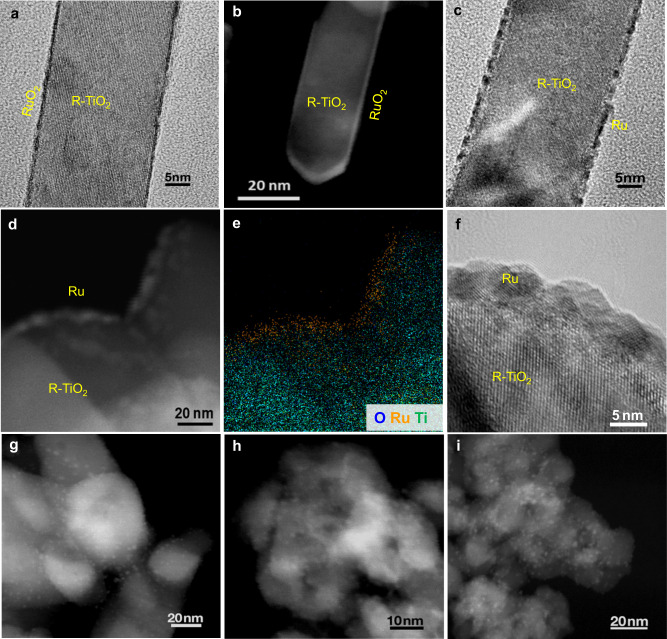
Structure characterizations of Ru/TiO_2_ catalysts. **a** TEM and **b** STEM images of RuO_2_ overlayers on R–TiO_2_ nanorods. **c** Ru/R–TiO_2_ reduced from RuO_2_/R–TiO_2_ by H_2_. **d** STEM, **e** elemental mapping, and **f** TEM images of Ru/R–TiO_2_–air–H_2_ after CO_2_ hydrogenation. STEM images of **g** Ru/R–TiO_2_–H_2_, **h** Ru/A–TiO_2_–air–H_2_, and **i** Ru/A–TiO_2_–H_2_ after reactions.

Given that R–TiO_2_ and A–TiO_2_ share the same chemical compositions, supports and catalysts were annealed and prepared following the same procedures, and reaction conditions were controlled the same, Ru–TiO_2_ interfacial interactions should dominate the opposite support effects. RuO_2_ shares the same lattice structure with R–TiO_2_, and their lattice misfit is less than 3.0%, thus RuO_2_ can form epitaxial overlayers on R–TiO_2_ with zero contact angles^39^. Figure 2a, b and Supplementary Fig. 5 present transmission electron microscopy (TEM) and scanning TEM (STEM) images of RuO_2_/R–TiO_2_ prepared by annealing RuCl_3_–R–TiO_2_ mixture at 400 °C in air. RuO_2_ encapsulates R–TiO_2_ nanorods as epitaxial overlayers and forms core–shell structures. Such epitaxial structures can enhance the activity and stability of RuO_2_ in catalytic oxidation reactions, such as Deacon reaction^44^. In contrast, RuO_2_ supported on A–TiO_2_ are NPs rather than epitaxial overlayers due to their different lattice types (Supplementary Fig. 6). Therefore, RuO_2_ shows a much higher interfacial compatibility with R–TiO_2_ than A–TiO_2_—this intrinsically determines their opposite support effects on the catalytic performances of Ru NPs.

The different interfacial compatibilities modify the surface states of Ru NPs on TiO_2_ supports first. For Ru/R–TiO_2_–air–H_2_, Ru can still partly show epitaxial structures after H_2_ reduction (Fig. 2c). In particular, after CO_2_ hydrogenation reaction, Ru presents as flat NPs on Ru/R–TiO_2_–air–H_2_ as shown by the images of STEM (Fig. 2d), elemental mapping (Fig. 2e), and high-resolution TEM (Fig. 2f). In contrast, for Ru/R–TiO_2_–H_2_, Ru/A–TiO_2_–H_2_, and Ru/A–TiO_2_–air–H_2_, Ru exists as NPs with average sizes around 2.0–3.0 nm after reactions (Fig. 2g, i, Supplementary Fig. 7). Specifically, size distributions of Ru nanoparticles on Ru–TiO_2_–air–H_2_ and Ru–TiO_2_–H_2_ are 2.4 ± 0.4 nm and 2.5 ± 0.5 nm, respectively (Supplementary Fig. 7). The high interfacial compatibility can intrinsically increase interfacial coupling strength, and modify the chemical and surface states of Ru species, MSI modes, and catalytic performances of Ru/TiO_2_ catalysts.

### Interfacial bonding states of Ru/TiO_2_ catalysts

**Fig. 3:**
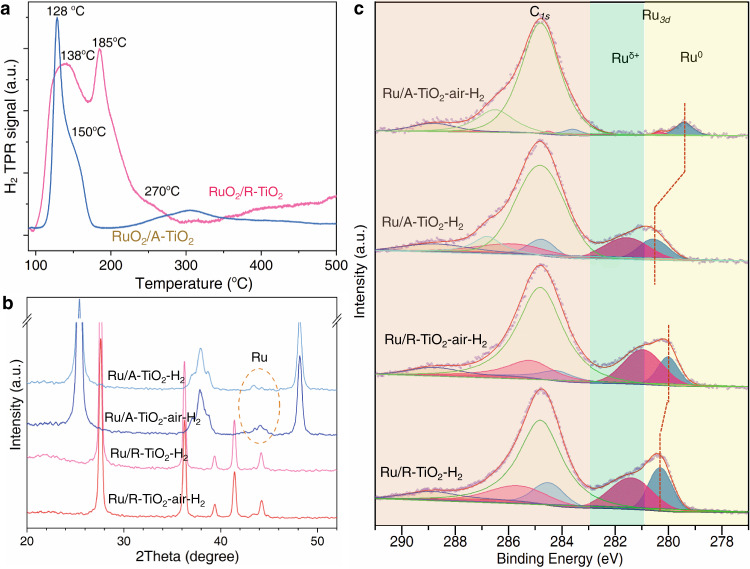
Characterizing the chemical states of Ru species on TiO_2_ supports. **a** H_2_-TPR of RuO_2_/A–TiO_2_ and RuO_2_/R–TiO_2_. **b** XRD patterns and **c** XPS spectra of Ru/TiO_2_ catalysts.

We use H_2_ temperature-programmed reduction (H_2_-TPR) to probe the effects of interfacial compatibility on Ru–TiO_2_ coupling strengths (Fig. 3a). H_2_-TPR is an effective method to characterize the reducibility of oxides and their interfacial interaction strengths with supports^37,45^. We prepared RuO_2_/TiO_2_ materials by annealing RuCl_3_–TiO_2_ mixtures at 400 °C in air. H_2_-TPR results show that RuO_2_/A–TiO_2_ can be reduced between 110 °C and 175 °C; the peaks at 128 °C, 150 °C, and 300 °C correspond to surface RuO_2_, interfacial RuO_x_, and surface A–TiO_2_ species, respectively. While RuO_2_/R–TiO_2_ can be reduced between 100 °C and 290 °C, and shows three states at 138, 185, and 270 °C, corresponding to surface RuO_2_, interfacial RuO_x_ species^45^. The higher reduction temperature indicates that RuO_x_ is more stable on R–TiO_2_, which further confirms the stronger interfacial coupling due to matched lattices. Furthermore, X-ray diffraction (XRD) patterns of Ru/A–TiO_2_ display clear Ru signals, while no Ru peaks appear on Ru/R–TiO_2_–air–H_2_ (Fig. 3b, Supplementary Fig. 8). The results indicate higher dispersions of Ru on R–TiO_2_, agreeing with TEM results.

We use X-ray photoelectron spectroscopy (XPS) to probe the chemical states of Ru. Figure 3c presents Ru-*3d* and C-*1s* lines of 2% Ru/TiO_2_ catalysts after catalytic reactions, in which C-*1s* is set at 284.8 eV. Ru-*3d*_3/2_ spectra show both metallic (Ru^0^) and oxidized (Ru^*δ*+^) states, in which oxidized states mainly locate at Ru–TiO_2_ interfaces. Ru^*δ*+^/Ru^0^ ratio is 1.3 for Ru/R–TiO_2_–H_2_ and 1.5 for Ru/A–TiO_2_–H_2_ (Table 1). The approximately identical Ru^*δ*+^/Ru^0^ ratios suggest that directly-reduced Ru NPs show similar interactions with R–TiO_2_ and A–TiO_2_, agreeing with their catalytic performances. However, pre-annealing in air can dramatically alter the states of Ru. Ru^*δ*+^/Ru^0^ ratio increases to 2.2 for Ru/R–TiO_2_–air–H_2_, indicating increased interfacial contacts. This trend can be reproduced on other Ru/R–TiO_2_ catalysts of varied R–TiO_2_ supports and loading ratios of Ru (Supplementary Figs. 9 and 10, Supplementary Table 1). In contrast, Ru^*δ*+^/Ru^0^ ratio decreases to 0.2 for Ru/A–TiO_2_–air–H_2_, and Ru^*δ*+^ state is very weak. The opposite trends of Ru^*δ*+^/Ru^0^ ratio also agree with their opposite catalytic performances.

**Table 1:** XPS results of 2% Ru/TiO_2_ catalysts.

Sample	Binding energy (eV)		Ru^*δ*+^/Ru^0^ radio
	Ru^*δ*+^	Ru^0^	
Ru/R–TiO_2_–air–H_2_	281.0	280.0	2.2
Ru/R–TiO_2_–H_2_	281.4	280.3	1.3
Ru/A–TiO_2_–air–H_2_	280.3	279.4	0.2
Ru/A–TiO_2_–H_2_	281.5	280.5	1.5

Another feature of Ru/A–TiO_2_–air–H_2_ lies in the shift of Ru^0^-*3d*_5/2_ from 280.5 to 279.4 eV, a binding energy even lower than that of Ru foil (280.1 eV, Supplementary Fig. 11). This phenomenon is usually ascribed to the occurrence of SMSI effect^46^. This can also be supported by the catalytic performances. For CO_2_ hydrogenation, SMSI and catalyst sizes can highly affect activity and selectivity^5,21,22,45^. Both SMSI effect and size reduction can convert the product from CH_4_ to CO^43,47,48^. For example, Paraskevi reported that 3-nm Ru NPs showed the highest turnover frequency (TOF) on TiO_2_, and bigger NPs favor CH_4_^5^. In our system, pre-annealing increases the size of Ru NPs on A–TiO_2_ from 2.1 nm to 2.8 nm, but the activity and CH_4_ selectivity both dramatically decrease. The results indicate that SMSI effect should account for this change, because SMSI can generally decrease the activity and selectivity of CO_2_ methanation^18^. For example, for Rh/TiO_2_, adsorbates can induce SMSI to decrease the activity and selectivity for CO_2_ methanation; Ru/TiO_2_ also shows SMSI effect in reduction reactions^18,49,50^. Therefore, varied interfacial compatibilities can vary MSI modes of Ru/TiO_2_–air–H_2_ catalysts: Ru/R–TiO_2_–air–H_2_ shows enhanced interfacial coupling bridged by RuO_x_ layers, while Ru/A–TiO_2_–air–H_2_ shows SMSI effect.

### Surface atomic states of Ru NPs probed with CO-DRIFTS

**Fig. 4:**
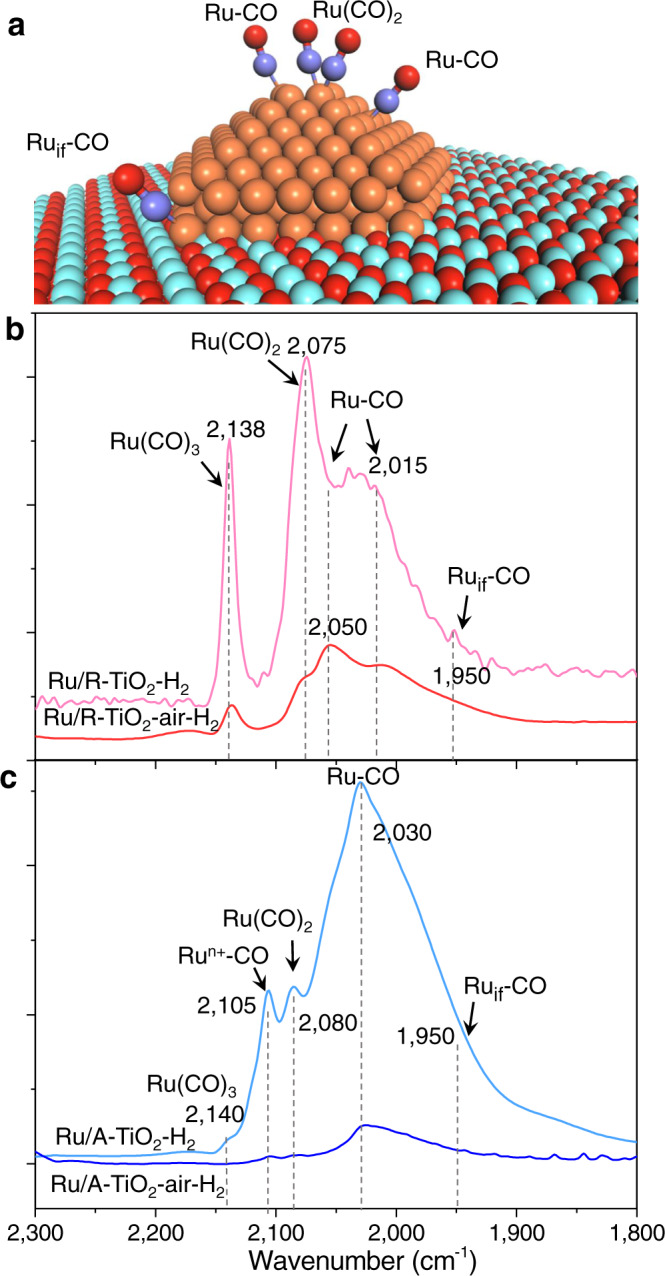
Probing surface atomic states of Ru NPs with CO-DRIFTS at 25 °C. **a** Adsorption configurations of CO on Ru/TiO_2_ catalysts. **b** and **c** CO-DRIFTS of Ru/TiO_2_ catalysts.

Different MSI modes can vary the exposed surface atomic states of Ru NPs. We characterize surface Ru sites using diffuse-reflectance infrared Fourier transform spectroscopy (DRIFTS) at 25 °C with CO as the probe (Fig. 4). Figure 4a schemes the possible adsorption configurations of CO on Ru/TiO_2_. For Ru/A–TiO_2_–H_2_, 2140 and 2080 cm^−1^ result from multi-carbonyl-adsorption modes of CO on Ru sites with low coordination numbers (Ru(CO)_*x*_, *x* = 2, 3), while the modes appear at 2138 cm^−1^ and 2075 cm^−1^ for Ru/R–TiO_2_–H_2_^51^. The broad peaks from 1900 to 2070 cm^−1^ result from top-absorption modes of CO on Ru NPs (Ru–CO) and at the interface between Ru and TiO_2_ (Ru_if_–CO)^41,42^. For Ru/R–TiO_2_–air–H_2_, the proportion of 1950 derived from Ru_if_–CO was higher than directly reduced, agreeing with the XPS results. The bands at 2030 and 2015 cm^−1^ belong to CO linearly adsorbed on Ru surfaces with A–TiO_2_ and R–TiO_2_ (Ru–CO), while the peak at 2105 cm^−1^ on A–TiO_2_ corresponds to Ru nanoclusters (Ru^n+^–CO). These Ru nanoclusters catalyze the formation of CO (<2%) above 240 °C (Fig. 1c).

After pre-annealing, the total intensities decrease, and the relative intensities of the peaks also change. In particular, Ru(CO)_*x*_ mode on low coordinated Ru (Ru_LC_) sites decreases for Ru/R–TiO_2_–air–H_2_, while it almost disappears for Ru/A–TiO_2_–air–H_2_. These changes mean that the ratios of Ru_LC_ sites decrease after annealing. Usually, the ratio of low coordinated surface atoms decreases with increased particle sizes. The result of Ru/A–TiO_2_–air–H_2_ is consistent with this trend, as shown by increased sizes of Ru NPs from 2.1 nm to 2.8 nm (Supplementary Fig. 7). The projected sizes of Ru NPs for Ru/R–TiO_2_–air–H_2_ and Ru/R–TiO_2_–H_2_ are 2.4 ± 0.4 nm and 2.5 ± 0.5 nm (Supplementary Fig. 7), which are almost the same. This suggests that the decreased ratios of Ru_LC_ sites did not result from increased particle sizes. TEM images show that Ru NPs display flatter shapes for Ru/R–TiO_2_–air–H_2_, owing to the stronger affinity of Ru with rutile supports bridged by interfacial RuO_x_ species (Fig. 2d–f). The greater curvature radius of flatter particles can lead to more ordered arrangement of surface atoms and increase the number of surrounding atoms. We also characterize the metal dispersion of Ru NPs with CO pulse adsorption. Metal dispersions of Ru (*D*_*co*_) are 33.6% and 31.1% for Ru/R–TiO_2_–air–H_2_ and Ru/R–TiO_2_–H_2_, respectively, which are almost the same (Supplementary Table 2). The results thus indicate that size and surface area does not play critical roles in enhancing the catalytic performances of Ru NPs on R–TiO_2_ supports. Therefore, such different surface atomic configurations of Ru nanoparticles directly modify their catalytic performances in CO_2_ methanation.

### Reaction mechanisms probed with operando FTIR

**Fig. 5:**
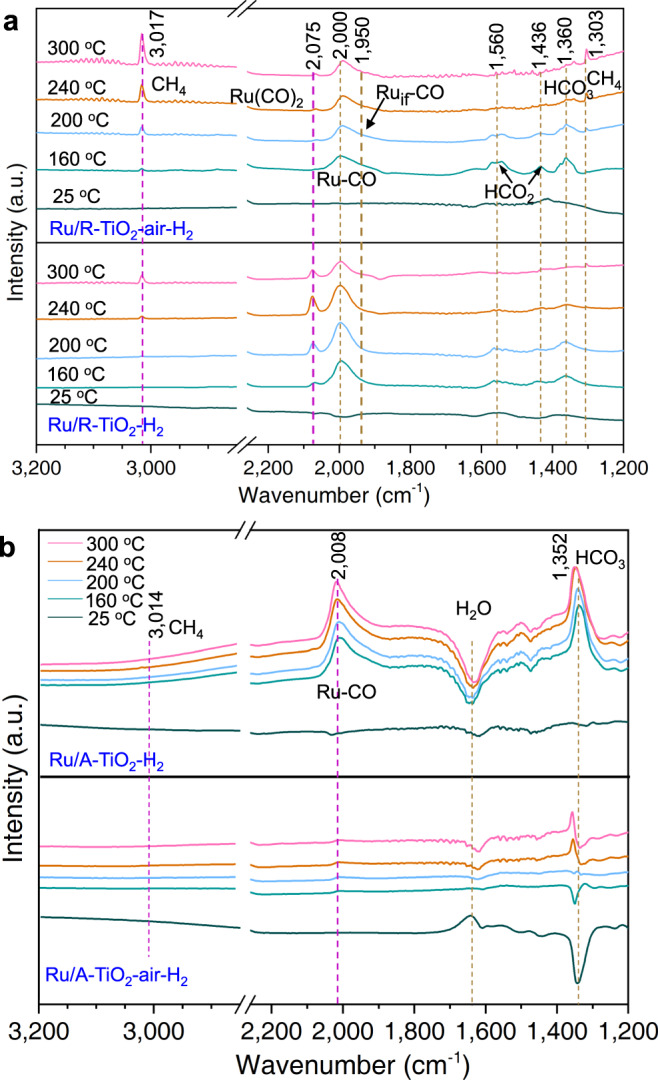
Revealing reaction mechanisms of CO_2_ hydrogenation on Ru/TiO_2_ catalysts with operando FTIR from 25 °C to 300 °C. **a** Reaction mechanisms on Ru/R–TiO_2_–H_2_ and Ru/R–TiO_2_–air–H_2_. **b** Reaction mechanisms on Ru/A–TiO_2_–H_2_ and Ru/A–TiO_2_–air–H_2_.

We further use operando Fourier transform-infrared spectroscopy (operando FTIR) to reveal CO_2_ hydrogenation mechanisms on these Ru/TiO_2_ catalysts (Fig. 5). The measurements were performed from 25 to 300 °C in 20-sccm gas flows of 60 vol%H_2_/15 vol%CO_2_/25 vol% Ar. For Ru/R–TiO_2_ (Fig. 5a), the absorptions at 3017 and 1303 cm^−1^ result from C to H bonds of CH_4_, 1436 and 1560 cm^−1^ from adsorbed formate species (*HCO_2_), 1950, 2075 from adsorbed CO species (*CO), and 1360 cm^−1^ from adsorbed carbonate (*HCO_3_)^5,41,42,52^.

At 25 °C, both Ru/R–TiO_2_–air–H_2_ and Ru/R–TiO_2_–H_2_ show weak adsorption peaks, but the reactions can obviously occur above 160 °C. At 160 °C, *HCO_3_, *HCO_2_, and *CO appear on both catalysts, meaning CO_2_ is first activated as *HCO_3_ and *HCO_2_, and further reduced into *CO^8,41^. The difference lies in 2075 and 3017 cm^−1^. At 160 °C, CH_4_ appears on Ru/R–TiO_2_–air–H_2_ but not on Ru/R–TiO_2_–H_2_, indicating that Ru/R–TiO_2_–air–H_2_ is more active for CO_2_ methanation. This agrees with the enhanced catalytic results. Many researches have figured out that *CO and formate are two possible intermediates in thermal CO_2_ hydrogenation reactions. In our results, stepwise-increasing reaction temperatures can lead to similar changes of *HCO_2_ on the two samples (Fig. 5a). This suggests that formate is not likely the intermediate, or at least not linked to the distinctly different activity on rutile. Some previous reports have also concluded that *CO hydrogenation is the rate-determining step in the CO_2_ hydrogenation on Ru/TiO_2_^41^. Two catalysts show different adsorption modes of ^*^CO, more obvious multicarbonyl Ru(CO)_*n*_ species at 2070 cm^−1^ on Ru/R–TiO_2_–H_2_, which adsorbed on Ru_LC_ of the surfaces of Ru nanoparticles. It is inactive at low temperatures because H_2_ cannot effectively reduce it at low temperatures^51^.

For Ru/A–TiO_2_ catalysts, the modes at 3014 cm^−1^ result from C to H bonds of CH_4_, 2008 cm^−1^ from *CO, and 1352 cm^−1^ from *HCO_3_ (Fig. 5b). This suggests that the reaction routes are the same with Ru/R–TiO_2_, agreeing with our catalytic results in fixed-bed reactors. While the reversal peaks at 1644 and 1344 cm^−1^ originate from the desorption of *H_2_O, *OH, and *H on catalyst surfaces during the reaction^53^. For Ru/A–TiO_2_–air–H_2_, the intensity at 2008 cm^−1^ sharply drops compared with Ru/A–TiO_2_–air–H_2_, in line with CO-DRIFTS results in Fig. 4c. The highly decreased Ru–CO mode at 2008 cm^−1^ indicates reduced exposure of surface Ru sites, which further supports the occurrence of SMSI, and agrees with the low catalytic activity (Fig. 1b). While SMSI effect can effectively convert the product from CH_4_ into CO^12,17,18,48^.

## Discussion

Our results show that rutile and anatase TiO_2_ supports can dramatically modify the morphology, surface atomic configuration, MSI mode, and catalytic performances of Ru catalysts for CO_2_ hydrogenation reaction, although they share the same chemical compositions. Ru NPs adhere stronger with R–TiO_2_ than A–TiO_2_, which disagrees with the trend that more active surface-oxygen atoms lead to stronger interfacial adhesion^28,29^. Instead, this confirms that interfacial compatibility plays critical roles in controlling the metal-support adhesion strength and MSI modes of Ru/TiO_2_ catalysts.

**Fig. 6:**
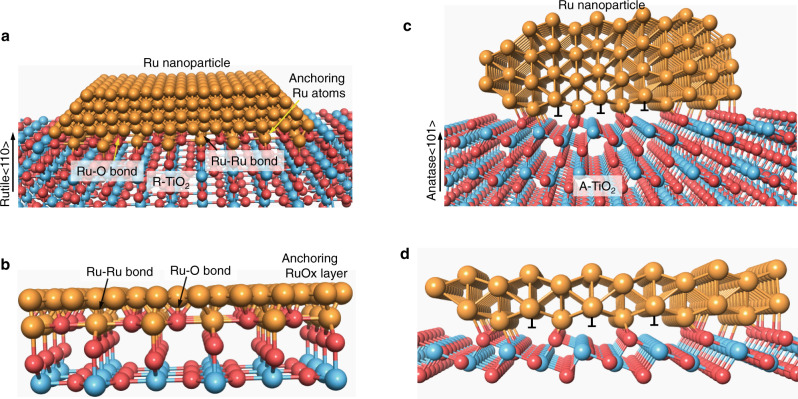
Atomic schemes showing the effects of interfacial compatibility on Ru/TiO_2_ adhesion strengths. **a** Scheme of epitaxial dispersion of Ru nanoparticle on rutile TiO_2_(110) surface. **b** Interfacial Ru–O and Ru–Ru bonds in anchoring RuO_x_ layer on R–TiO_2_. **c** Scheme of Ru nanoparticle on anatase TiO_2_ (101) surface. **d** Scheme of interfacial of bonds and defects of Ru with A–TiO_2_.

For Ru/TiO_2_ catalysts, RuO_2_ shares the same lattice structure with R–TiO_2_, thus, annealing RuCl_3_–R–TiO_2_ precursor can incorporate Ru atoms into the surface lattices of R–TiO_2_ to form epitaxial RuO_x_ species (Fig. 6a, b). Such interfacial RuO_x_ species can act as anchoring layers to strongly bind Ru nanoparticles onto R–TiO_2_ supports, which yields flat shapes with low contact angles and larger curvatures. This morphology can decrease the ratio of undercoordinated surface sites (Fig. 6a), and further modifies CO adsorptions and reaction routes. At the atomic scale, Ru^δ+^ atoms can adequately occupy Ti sites, thus bonding to R–TiO_2_ substrate with maximized bonding strength and density. While Ru^0^ atoms of Ru NPs can further bond to such Ru^δ+^ sites through Ru–Ru metallic bonds (Fig. 6b). This kind of binding features can minimize interfacial defects of strain, dislocation, and vacancies, thus can further highly enhance metal-support adhesion and suppress interfacial phase separation.

The lattice type of A–TiO_2_ (I4_1_/amd) is different from that of RuO_2_ (P4_2_/mnm), thus their interfacial atomic configurations do not match. Such misfit interfaces can form defects like edge dislocation and vacancy, which reduces interfacial adhesion and stability. Figure 6c–d schemes atomic interfacial contact of Ru nanoparticle on A–TiO_2_ (101) surface. Some Ru atoms bond to surface oxygen atoms through Ru–O bonds, but their bonding lengths and strengths vary, depending on their atomic positions. Moreover, some atoms cannot effectively bond to surface-oxygen atoms due to misfit positions, and these sites form dislocations (Fig. 6d). Therefore, Ru NPs weakly adhere A–TiO_2_ surfaces, and appear as spherical particles. This morphology yields more undercoordinated Ru sites. While the higher reducibility of surface-oxygen atoms can further drive the occurrence of SMSI effect, which further modifies CO adsorption, catalytic activity, and selectivity.

In summary, we have demonstrated that interfacial compatibility can critically control the interfacial coupling strength, surface atomic configurations, MSI modes, and catalytic performances of Ru/TiO_2_ catalysts by varying interfacial adhesion strengths. For CO_2_ methanation, enhanced interfacial coupling of Ru/R–TiO_2_ can increase catalytic activity and CH_4_ selectivity, while SMSI effect on Ru/A–TiO_2_ can highly decrease catalytic activity and convert the product from CH_4_ into CO. This is because Ru NPs can strongly adhere to R–TiO_2_ supports and form flat particles with larger curvatures, in which interfacial RuO_x_ species act as anchoring layers with R–TiO_2_; while Ru/A–TiO_2_ show classic SMSI effect due to lattice misfit and higher reducibility of surface oxygen atoms. Therefore, interfacial compatibility is a critical structural feature that can intrinsically modulate MSI modes and catalytic performances, which might be realized through designing interfacial atomic configurations, introducing anchoring layers, thermal annealing, and oxidation treatments. This work paves the way to improve catalytic performances through engineering interfacial compatibilities between metal NPs and supports.

## Methods

### Chemicals

Titanium tetrachloride (TiCl_4_, 99.5%), titanium trichloride (15.0~20% TiCl_3_ basis in 30% HCl), TiO_2_ (anatase, 25 nm), sodium hydroxide (NaOH, >98%), and sodium chloride (NaCl, 99.6%) were purchased from Shanghai Aladdin Biochemical Technology Co., Ltd. Ethanol (>99.7%), hydrochloric acid (HCl, 36–38%), and nitric acid (HNO_3_, 68%) were purchased from Sinopharm Chemical Reagent Beijing Co., Ltd. Ruthenium trichloride hydrate (RuCl_3_·xH_2_O, 37.5–41% Ru) and titanium nitride (TiN, 99%) were purchased from Beijing Innochem Science & Technology Co., LTD.

### Syntheses of rutile-type TiO_2_ nanorods

R–TiO_2_ nanorods were prepared using three methods:

1. TiCl_4_ (2.5 mL) was slowly added into ice water (30 mL) under vigorous stirring. After being stirred for 15 min, the solution was transferred into a 40-mL Teflon-lined stainless-steel autoclave. Then the solution was heated at 170 °C for 14 h in an oven. After cooling down to room temperature, the products were collected by centrifugation and washed 4 times with deionized water, and dried at 60 °C for 12 h^54^.
2. TiCl_3_ (5 mL) was first mixed with 30 mL of 1.0 M NaCl solution. After being transferred into a 40 mL Teflon-lined stainless-steel autoclave, the solution was heated at 200 °C for 6 h.
3. TiN (0.5 g) was dispersed into 30 mL of 4.0 M HNO_3_ solution, then was transferred into a 40 mL Teflon-lined stainless-steel autoclave. The mixture was heated at 180 °C for 24 h^55^.

### Syntheses of Ru/TiO_2_ catalysts

All Ru/TiO_2_ catalysts were prepared following the same procedure, in which only TiO_2_ supports were changed. (1) TiO_2_ supports were annealed in air at 500 °C for 10 h to stabilize their surfaces. (2) Then 1.0 g of the annealed TiO_2_ supports and a certain amount of RuCl_3_ were mixed in 10 mL of H_2_O under sonication for 30 min. (3) The mixtures were rapidly frozen with liquid nitrogen, and further dried in a freeze drier. (4) As-obtained powders were calcined at 400 °C for 4 h in air or directly reduced with H_2_ to prepare Ru/TiO_2_ catalysts.

### Characterization

Power X-ray diffraction (XRD) data were collected on a Bruker D8 diffractometer using Cu Kα radiation (1.5418 Å), which was operated at 40 kV and 40 mA with a scanning rate of 6 degree/min. High-resolution transmission electron microscopy (HRTEM), high-angle annular dark-field scanning transmission electron microscopy (HAADF-STEM), and element mapping were performed on FEI Tecnai F30 transmission electron microscope (TEM) under an acceleration voltage of 300 kV. Size distributions of Ru nanoparticles were obtained through measuring at least 100 particles. X-ray photoelectron spectroscopy (XPS) data were collected on Thermo Scientific ESCALAB 250Xi system using Al Kα line as the X-ray source. The spectra were calibrated by with C1s peak at 284.8 eV.

#### H_2_-TPR

H_2_ temperature-programmed reduction (H_2_-TPR) and CO pulse adsorption were performed on Autochem1 II 2920 instrument. Before TPR measurement, the samples were annealed in Ar at 300 °C for 60 min, then cooled down to 50 °C. The signals were recorded online with a thermal conductivity detector (TCD), as the reactor was heated to 800 °C at a heating rate of 10 °C /min under 10% H_2_–90% Ar flows.

#### DRIFT

In situ diffuse-reflectance infrared Fourier transform (DRIFT) spectra of CO adsorption were performed on Thermo Fisher Nicolet iS50 with a resolution of 4 cm^−1^ at 25 °C. Prior to CO adsorption, the sample was treated in H_2_ flow at 300 °C for 1 h and then cooled down. Prior to collecting the background spectrum, the sample was purged with Ar for 30 min. Then 5% CO/Ar flow (20 sccm) was introduced into the reactor until saturated adsorptions. DRIFT spectra were collected until no gas-phase CO could be detected with Ar purging.

#### Operando FT-IR study of reaction mechanisms

The measurements were performed on Thermo Fisher Nicolet iS50. Prior to collecting the spectra, the samples were treated in H_2_ flow at 300 °C for 1 h and then purging with Ar for 1 h. The background spectrum was collected, until the reactor cooling down to 25 °C. Subsequently, the feed gas (*n*(H_2_):*n*(CO_2_):*n*(Ar)=15:60:25) was introduced, then the IR spectra were collected at certain temperatures after being stabilized for 30 min.

### Catalytic test of CO_2_ hydrogenation

CO_2_ hydrogenation reaction was carried out in a fixed-bed reactor made of stainless steel. First, the catalysts were in situ reduced at 300 °C for 1 h with 25-sccm pure H_2_ before catalytic testing. After cooling down to 100 °C, the gas was switched to the reaction gas with molar ratio of *n*(H_2_):*n*(CO_2_):*n*(Ar) = 60:15:25. The reaction pressure was controlled at 1 atm, and the gas hourly space velocities (GHSVs) were 12,000 mL·g^−1^·h^−1^. The outgassing gas compositions were detected using an online gas chromatography (GC) equipped with a TCD detector after the reactions were stabilized for 25 min at specific temperatures.

The CO_2_ conversion (*X*CO_2_) was calculated according to the following equation:

2 $${X}_{{{{{{\rm{CO}}}}}}_{2}}=\frac{{n}_{{{{{\rm{in}}}}}}\left({{{{{{\rm{CO}}}}}}}_{2}\right)-{n}_{{{{{\rm{out}}}}}}({{{{{{\rm{CO}}}}}}}_{2})}{{{{n}}}_{{{{{\rm{in}}}}}}({{{{{{\rm{CO}}}}}}}_{2})}=1-\frac{{{{A}}}_{{{{{\rm{out}}}}}}({{{{{{\rm{CO}}}}}}}_{2})/{{{A}}}_{{{{{\rm{out}}}}}}({{Ar}})}{{{{A}}}_{{{{{\rm{in}}}}}}({{{{{{\rm{CO}}}}}}}_{2})/{{{A}}}_{{{{{\rm{in}}}}}}({{Ar}})}$$

where *n*_*i*n_(CO_2_) and *n*_out_(CO_2_) refer to the molar number of the CO_2_ before or after the reaction, respectively, the *A*_in_(CO_2_) and *A*_in_(*Ar*) refer to the chromatographic peak areas of the CO_2_ and Ar in the reaction gas, and the *A*_out_(CO_2_) and *A*_out_(*Ar*) refer to the GC area after reactions.

The reaction rate (*v*) was calculated following:

3 $$v=\frac{{{{{{\rm{GHSVs}}}}}}\times {X}_{{{{{{\rm{CO}}}}}}_{2}}\times 15 \% }{22400\times {{{{{{\rm{m}}}}}}}_{{{{{\rm{Ru}}}}}}}$$

where GHSVs refer to the gas hourly space velocities and *m*_Ru_ refers to the mass of the Ru. Under this condition, the products are CO and CH_4_, so the selectivity of CO and CH_4_ (*S*_CO_ and *S*CH_4_) meets

4 $${S}_{{{{{\rm{CO}}}}}}+{S}_{{{{{{\rm{CH}}}}}}_{4}}=1$$

5 $${S}_{{{{{\rm{CO}}}}}}={{{{{{\rm{f}}}}}}}_{{{{{{\rm{CO}}}}}}/{{{{{{\rm{CH}}}}}}}_{4}}\cdot \frac{{A}_{{{{{\rm{CO}}}}}}}{{A}_{{{{{{\rm{CH}}}}}}_{4}}}\cdot {S}_{{{{{{\rm{CH}}}}}}_{4}}$$

where the f_CO/CH4_ refers to the relative correction factors of CO–CH_4_ obtained by the calibrating gas; the *A*_CO_ and *A*_CH4_ refer to the chromatographic peak areas of CO and CH_4_, respectively.

### Reporting summary

Further information on research design is available in the Nature Research Reporting Summary linked to this article.

## Supplementary information

Supplementary Information

Peer Review File

Reporting Summary

## Data Availability

The data supporting the findings of the study are available within the paper and its Supplementary Information. Source data are provided with this paper in excel format. Source data are provided with this paper.

## Source data

Source Data
