# Supplementary material for: Interfacial compatibility critically controls Ru/TiO2 metal-support interaction modes in CO2 hydrogenation
Source: Nat Commun. 2022 Jan 17;13:327. doi: 10.1038/s41467-021-27910-4 (PMC8764066; doi:10.1038/s41467-021-27910-4)
Supplement: Supplementary file 1 — Supplementary Information [file 41467_2021_27910_MOESM1_ESM.pdf]

Supplementary Information

Interfacial compatibility critically controls Ru/TiO_2 metal-support interaction modes in CO_2 hydrogenation

Jun Zhou[1+], Zhe Gao[2+], Guolei Xiang[1*], Tianyu Zhai[1], Zikai Liu[1], Weixin Zhao[1], Xin Liang[1], Leyu Wang[1*]

1. State Key Laboratory of Chemical Resource Engineering, Beijing University of Chemical Technology, Beijing, 100029, P.R. China
2. State Key Laboratory of Coal Conversion, Institute of Coal Chemistry, Chinese Academy of Sciences, Taoyuan South Road 27, Taiyuan 030001, China

[+] These authors contributed equally

[*]To whom correspondence should be addressed:
xianggl@mail.buct.edu.cn; (G. X.)
lywang@mail.buct.edu.cn (L. W.)

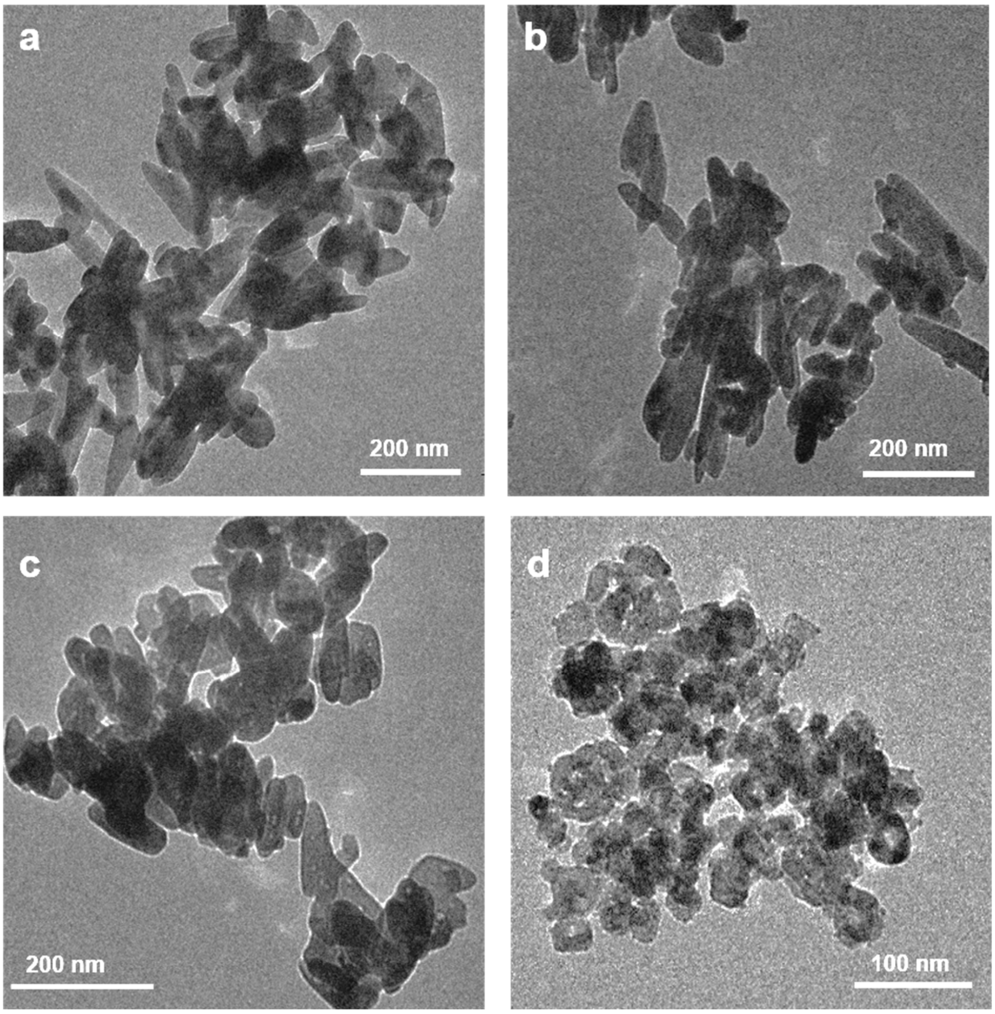

Supplementary Figure 1. TEM images of rutile-$TiCl_4$ (a), rutile-$TiCl_3$ (b), rutile-TiN (c) and anatase (d) after being annealed at 500 ^oC for 10 h.

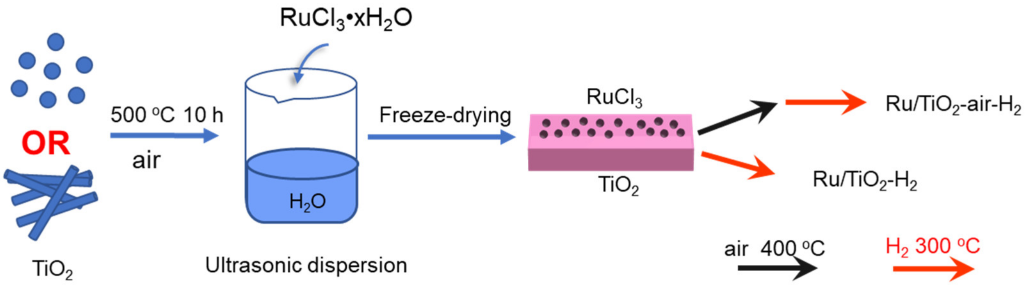

Supplementary Figure 2. Scheme of the synthetic procedures of Ru/TiO_2 catalysts.

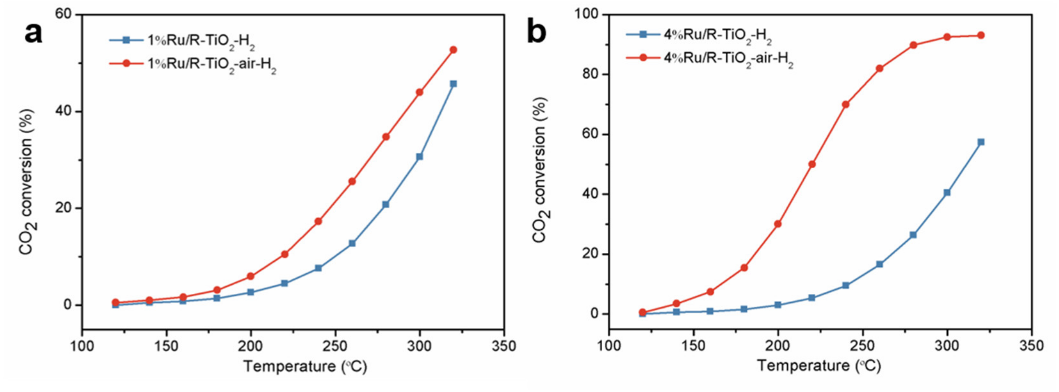

Supplementary Figure 3. Temperature-dependent CO_2 conversions of (a) 1%Ru/R-$TiO_2$and (b) 4%Ru/R-TiO_2.

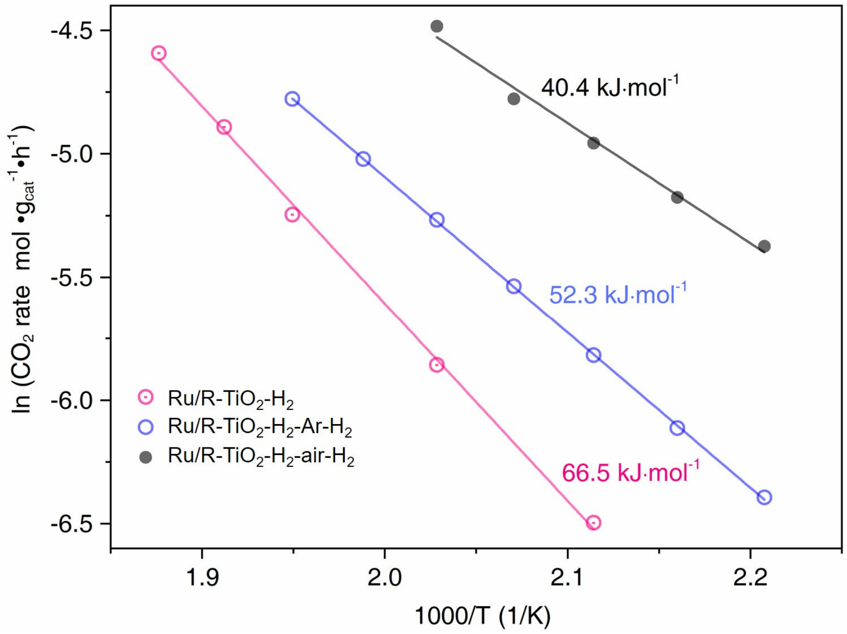

Supplementary Figure 4. Arrhenius plots of 2%Ru/R-TiO_2 catalysts treated in different procedures.

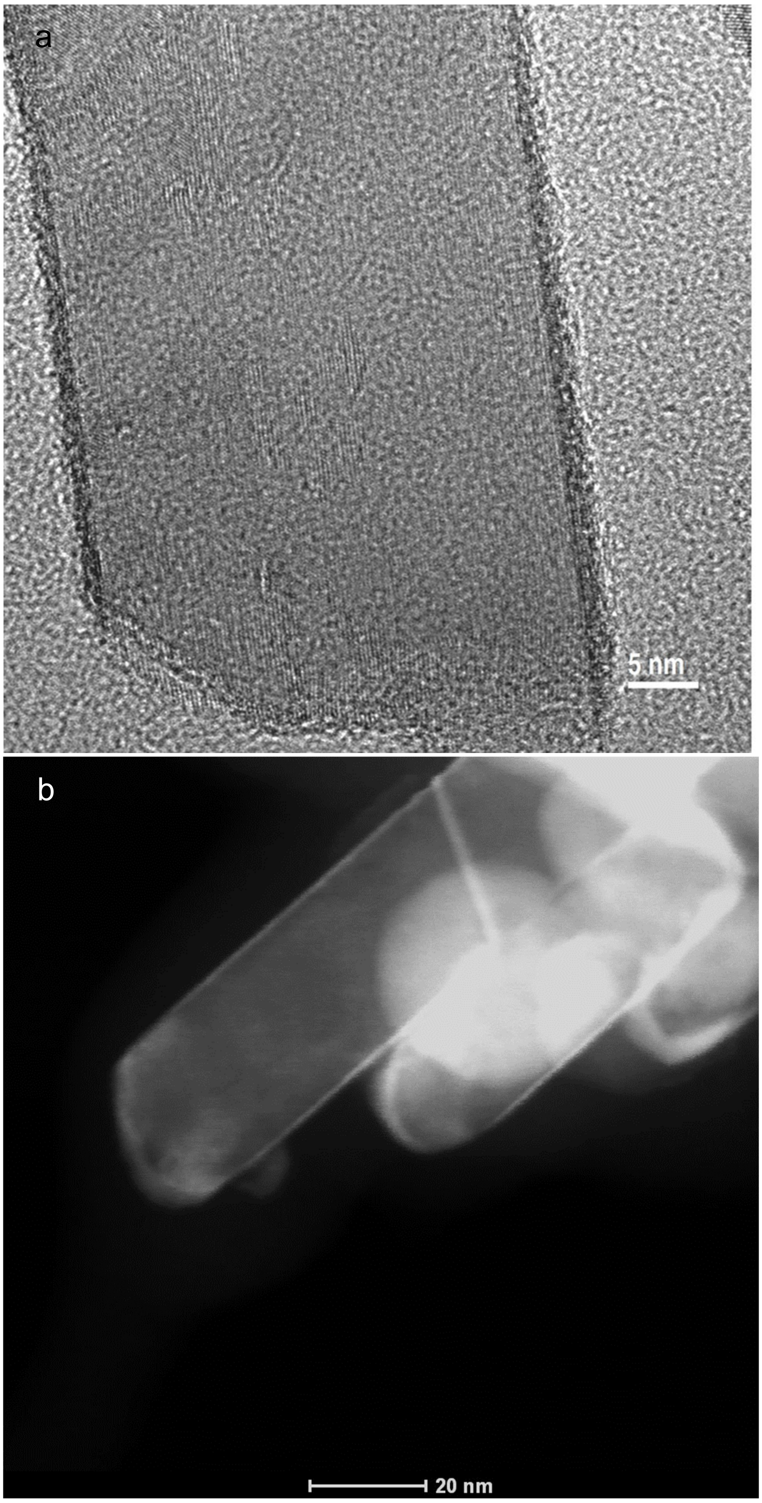

Supplementary Figure 5. TEM (a) and STEM (b) images of RuO_2 epitaxial layers on R-TiO_2 nanorod.

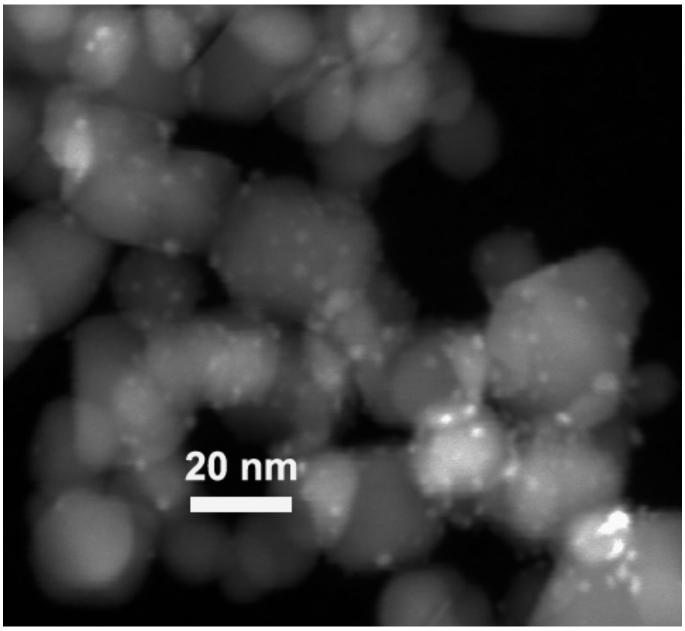

Supplementary Figure 6. STEM of RuO_2 on A-TiO_2.

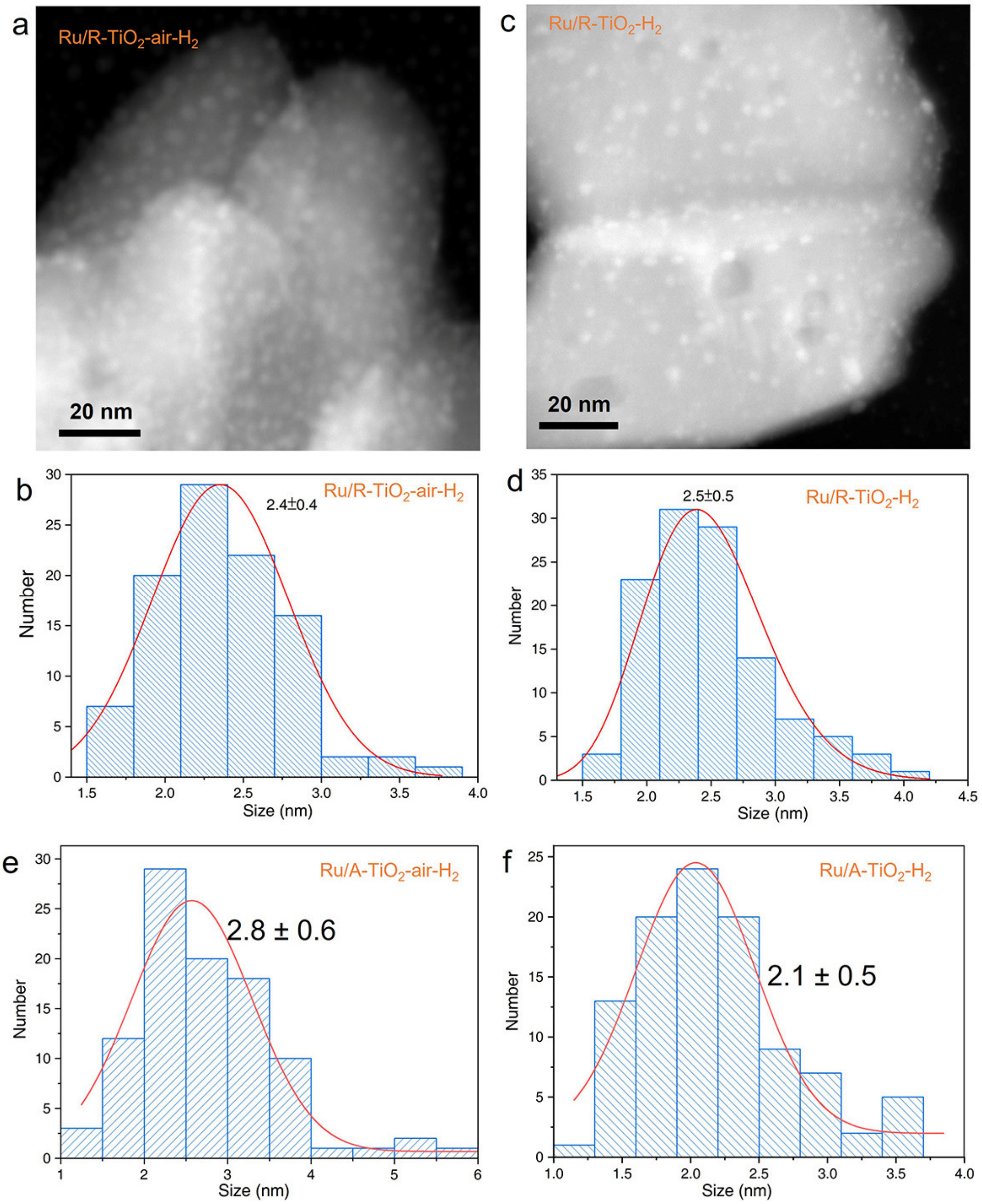

Supplementary Figure 7. Size distributions of 2%-Ru/TiO_2-H_2 catalysts. STEM images of (a) Ru/R-TiO_2-air-H_2 and (b) Ru/R-TiO_2-H_2 catalysts. Size distributions of Ru nanoparticles of (c) Ru/R-TiO_2-air-H_2, (d) Ru/R-TiO_2-H_2, (e) Ru/A-TiO_2-air-H_2, and (f) Ru/A-TiO_2-H_2 catalysts.

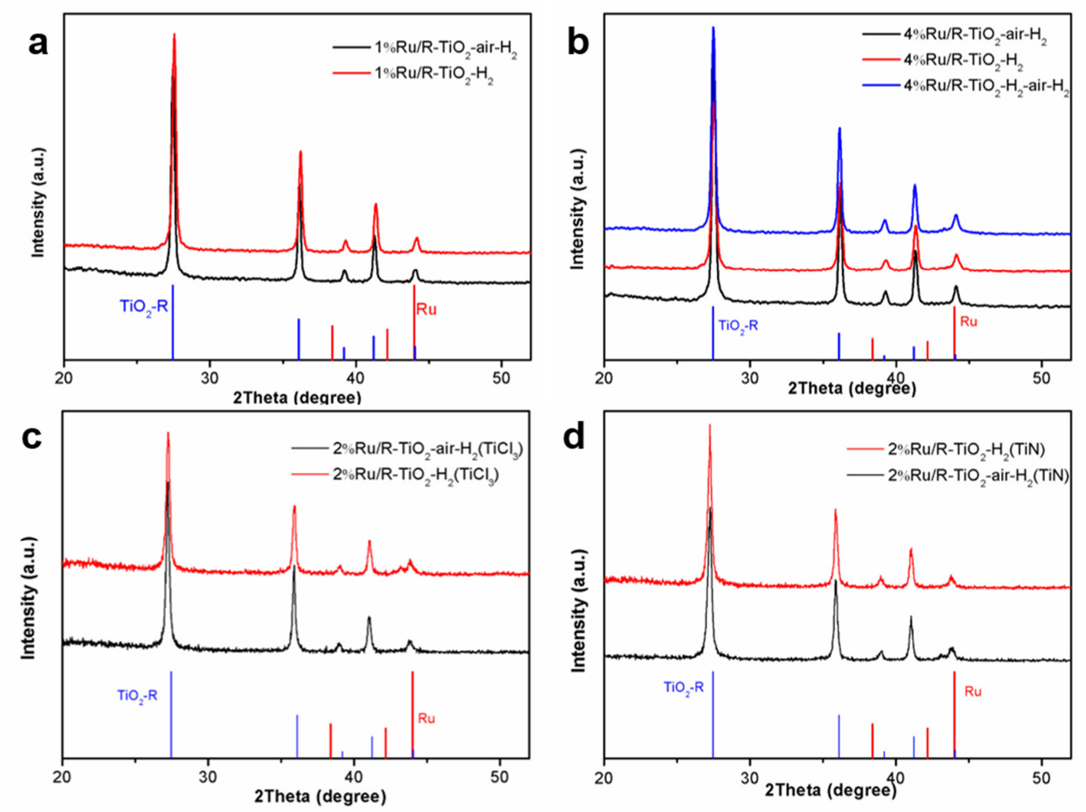

Supplementary Figure 8. XRD of 1%Ru/R-TiO_2 (a), 4%Ru/R-TiO_2(b), 2%Ru/R-TiO_2($TiCl_3$) (c) and 2%Ru/R-TiO_2(TiN) (d) after reaction

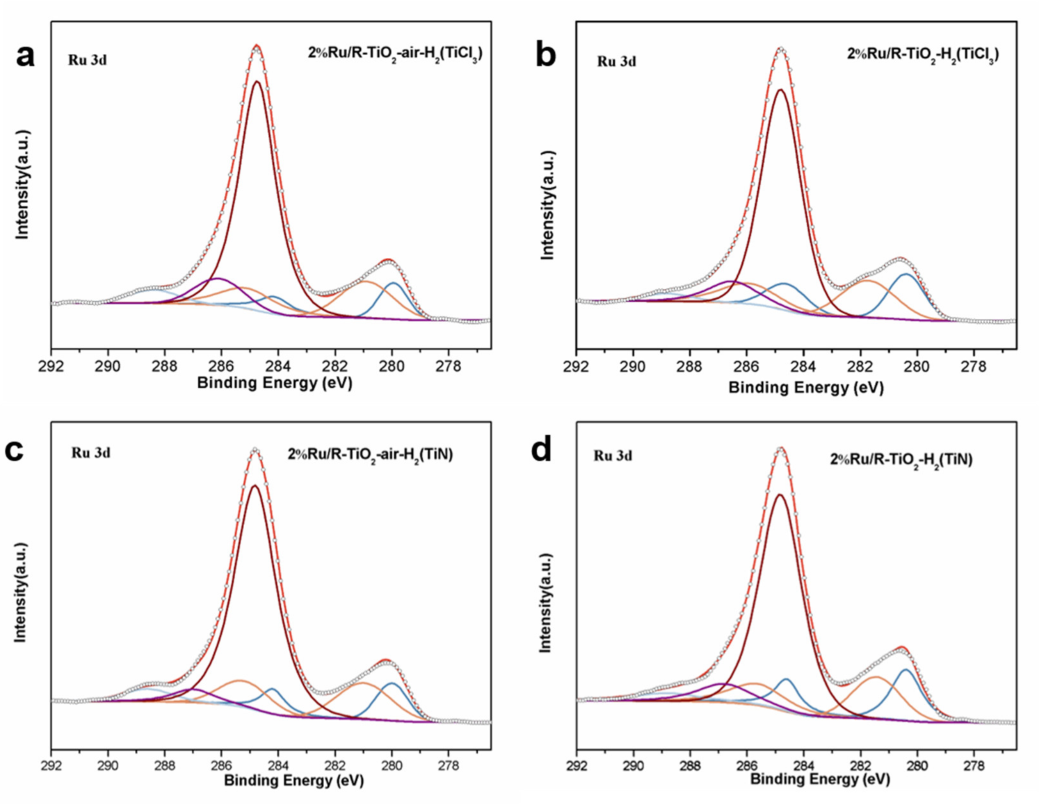

Supplementary Figure 9. XPS of 2%Ru/R-TiO_2-air-H2($TiCl_3$) (a), 2%Ru/R-TiO_2-H_2($TiCl_3$) (b), 2%Ru/R-TiO_2 -air-H_2(TiN) (c) and 2%Ru/R-TiO_2-H_2(TiN) (d) after reaction.

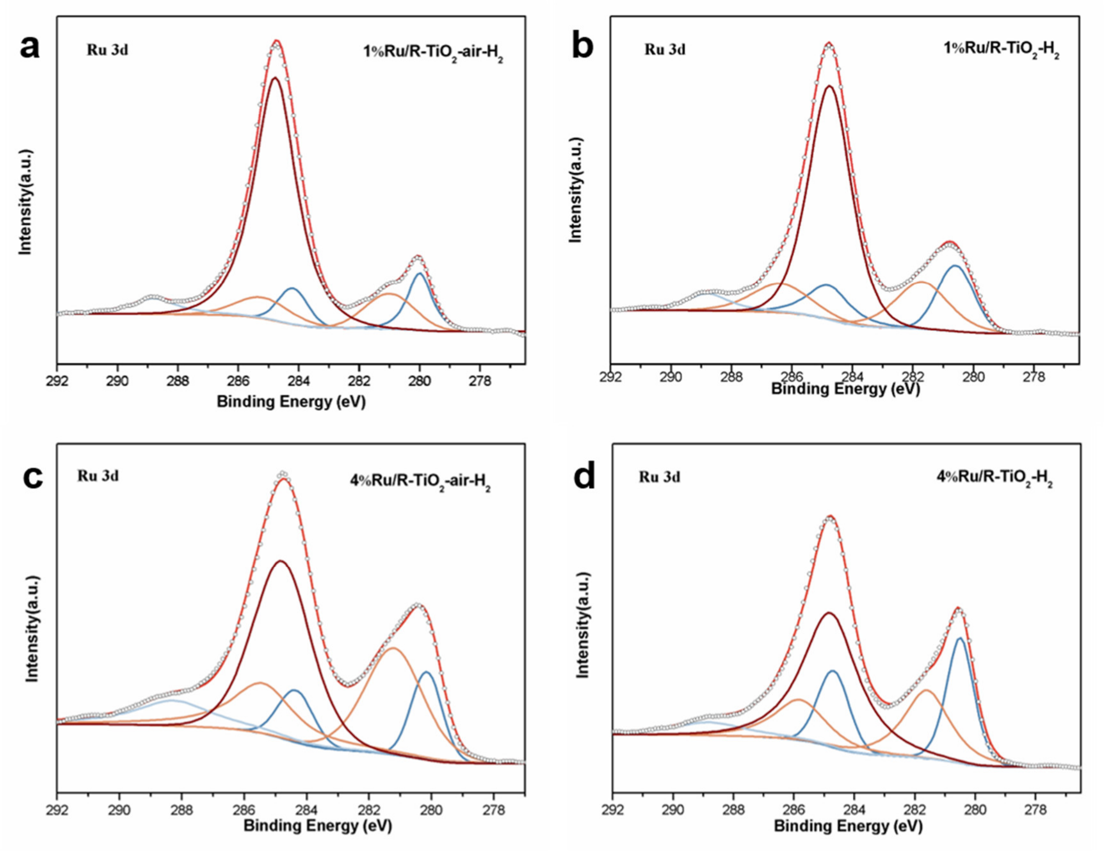

Supplementary Figure 10. XPS of 1%Ru/R-TiO_2-air-H_2(a), 1%Ru/R-TiO_2-H_2(b), 4%Ru/R-TiO_2-air-H_2(c) and 4%Ru/R-TiO_2-H_2(d) after reaction.

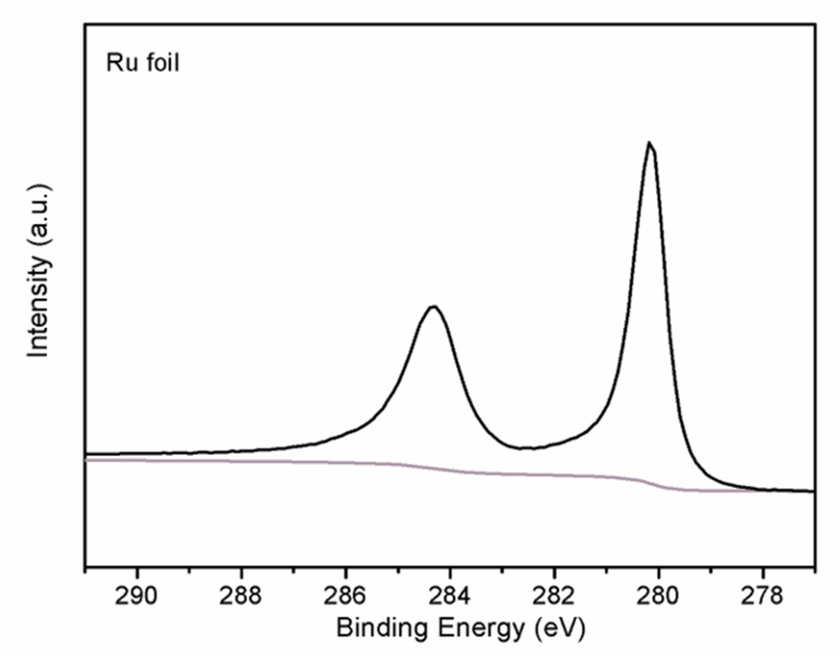

Supplementary Figure 11. XPS of Ru foil.

Supplementary Table 1. XPS fitting results of other catalysts.

Sample	Binding Energy [eV]		The radio of $Ru^{\delta+}/Ru^0$
	$Ru^{\delta+}$	Ru^0	
1%Ru/R-TiO_2-air-H_2	281.1	280.0	1.1
1%Ru/R-TiO_2-H_2	281.7	280.6	1.1
4%Ru/R-TiO_2-air-H_2	281.2	280.1	2.3
4%Ru/R-TiO_2-H_2	281.6	280.5	1.0
2%Ru/R-TiO_2-air-H_2($TiCl_3$)	281.1	280.0	1.8
2%Ru/R-TiO_2-H_2($TiCl_3$)	281.7	280.4	1.2
2%Ru/R-TiO_2-air-H_2(TiN)	280.9	279.9	1.9
2%Ru/R-TiO_2-H_2(TiN)	281.4	280.4	1.1

Supplementary Table 2. Metal dispersion of Ru on rutile calculated by CO pulse adsorption

Sample	Ru/R-TiO_2-air-H_2	Ru/R-TiO_2-H_2
D_{CO}(%)	33.64	31.1
